# Role of Pulse Pressure and Geometry of Primary Entry Tear in Acute Type B Dissection Propagation

**DOI:** 10.1007/s10439-016-1705-4

**Published:** 2016-08-10

**Authors:** Srikara V. Peelukhana, Yanmin Wang, Zachary Berwick, Jarin Kratzberg, Joshua Krieger, Blayne Roeder, Rachel E. Cloughs, Albert Hsiao, Sean Chambers, Ghassan S. Kassab

**Affiliations:** 1 California Medical Innovations Institute, San Diego, CA USA; 2 3DT holdings LLC, San Diego, CA USA; 3 Cook Medical Inc., Bloomington, IN USA; 4 Department of General Surgery, Qilu Hospital of Shandong University, Jinan, Shandong China; 5 Hôpital Cardiologique, Centre Hospitalier Régional Universitaire de Lille, Lille, France; 6 Department of Vascular Surgery and Division of Imaging Sciences, Guy’s and St Thomas’ Hospitals and King’s College London, London, UK; 7 Department of Radiology, UCSD, San Diego, CA USA

**Keywords:** Pulse pressure, Circumferential dissection, Axial dissection, Depth of dissection, Bench-models

## Abstract

The hemodynamic and geometric factors leading to propagation of acute Type B dissections are poorly understood. The objective is to elucidate whether geometric and hemodynamic parameters increase the predilection for aortic dissection propagation. A pulse duplicator set-up was used on porcine aorta with a single entry tear. Mean pressures of 100 and 180 mmHg were used, with pulse pressures ranging from 40 to 200 mmHg. The propagation for varying geometric conditions (%circumference of the entry tear: 15–65%, axial length: 0.5–3.2 cm) were tested for two flap thicknesses (1/3rd and 2/3rd of the thickness of vessel wall, respectively). To assess the effect of pulse and mean pressure on flap dynamics, the %true lumen (TL) cross-sectional area of the entry tear were compared. The % circumference for propagation of thin flap (47 ± 1%) was not significantly different (*p* = 0.14) from thick flap (44 ± 2%). On the contrary, the axial length of propagation for thin flap (2.57 ± 0.15 cm) was significantly different (*p* < 0.05) from the thick flap (1.56 ± 0.10 cm). TL compression was observed during systolic phase. For a fixed geometry of entry tear (%circumference = 39 ± 2%; axial length = 1.43 ± 0.13 cm), mean pressure did not have significant (*p* = 0.84) effect on flap movement. Increase in pulse pressure resulted in a significant change (*p* = 0.02) in %TL area (52 ± 4%). The energy acting on the false lumen immediately before propagation was calculated as 75 ± 9 J/m^2^ and was fairly uniform across different specimens. Pulse pressure had a significant effect on the flap movement in contrast to mean pressure. Hence, mitigation of pulse pressure and restriction of flap movement may be beneficial in patients with type B acute dissections.

## Introduction

Aortic dissection is a life-threatening disease with an incidence of 3.5–14 cases per 100,000 persons per year.^3^,^18^,^23^,^26^ It is characterized by a tear in the intima-media, where blood enters the layers of the aortic wall to create a false channel, known as the false lumen (FL) in addition to normal endothelialized channel known as the true lumen (TL). For reasons that have not been fully elucidated, an initial intimal-medial tear can propagate either antegrade or retrograde along the wall of the aorta, and may lead to complications including aneurysmal formation, frank rupture of the aortic wall, aortic valve regurgitation, coronary dissection, cardiac tamponade, hypotension/shock, end organ ischemia, and death.^10^,^13^,^14^,^18^,^23^,^26^ Major clinical risk factors associated with aortic dissection are hypertension, connective tissue disorder, or vasculitis.^3^,^10^,^26^

Based on the time elapsed since the onset of symptoms, dissections can be classified into acute (<2 weeks), subacute (2–6 weeks) and chronic (>6 weeks).^26^ Dissections are also classified based on their location. The Stanford classification forms the basis of acute surgical management, where dissections that involve the ascending aorta are termed Type A dissections while Type B dissections are those that do not involve the ascending aorta.^10^,^26^ A recent 17 year study has reported a decrease in Type A in-hospital mortality from 31 to 22% and no significant trend for the in-hospital mortality of type B dissections (12–14%).^25^

A full understanding of the biomechanical factors responsible for initiation and propagation of aortic dissections is lacking. Initial studies using hydro-dissection models^4^,^29^,^34^ on isolated porcine and human aortas reported mean propagation pressures in non-physiological ranges (197–579 mmHg). These studies used hydrostatic rather than pulsatile pressure, however, to propagate the dissection. Later studies utilized *in vitro* pulsatile flow conditions to understand the role of various hemodynamic factors in aortic dissection.^5^,^6^,^9^,^30^,^36^,^37^ In short, these studies used compliant material phantoms to study the pressure and flow characteristics in the TL and FL, in the presence of a static flap (e.g., gluing the flap to aortic wall representing a partial TL occlusion). Also, since biological tissue was not used, the presence of *in vivo* stretch was lacking in those studies.

The role of pulsatile flow on aortic propagation was suggested by Prokop *et al.*, 1970.^27^ This study correlated a value of (*dp*/*dt*)_max_ < 790 mmHg/s (in Tygon tubing) and <3800 mmHg/s (in Dog aortas) to dissection propagation. These values are rarely achieved in aorta, and the importance of this threshold has been questioned.^28^

In previous *in vitro* studies, the dynamic nature of dissection flap has not been explored. Clinically, the flap movement leads to significant variation in FL and TL areas during cardiac cycle.^2^,^12^,^21^,^38^ It is likely that this dynamic behavior is related to both the mean pressure and pulse pressure (defined as the difference between the systolic and diastolic pressure), which might play an important role in the initiation and propagation of dissections.^28^

Further, the de-cohesive energy responsible for flap propagation can be expressed as a function of the %circumference of the entry tear (ratio of the circumference of the tear to the circumference of the vessel), axial length of the tear, depth of the flap, and the pulse pressure. Hence, the objective is to elucidate the relation between the geometry of the dissection flap and the hemodynamics of the aorta. We *hypothesize* that the hemodynamic (e.g., pulse pressure) and geometric (e.g., degree of circumferential and axial dissection as well as the depth of dissection) parameters affect the propensity to propagation of dissection. To test these hypotheses, a pulsatile flow-loop was created with porcine aorta in a simulated type B dissection.

## Materials and Methods

### Experimental Set-Up

**Figure 1:**
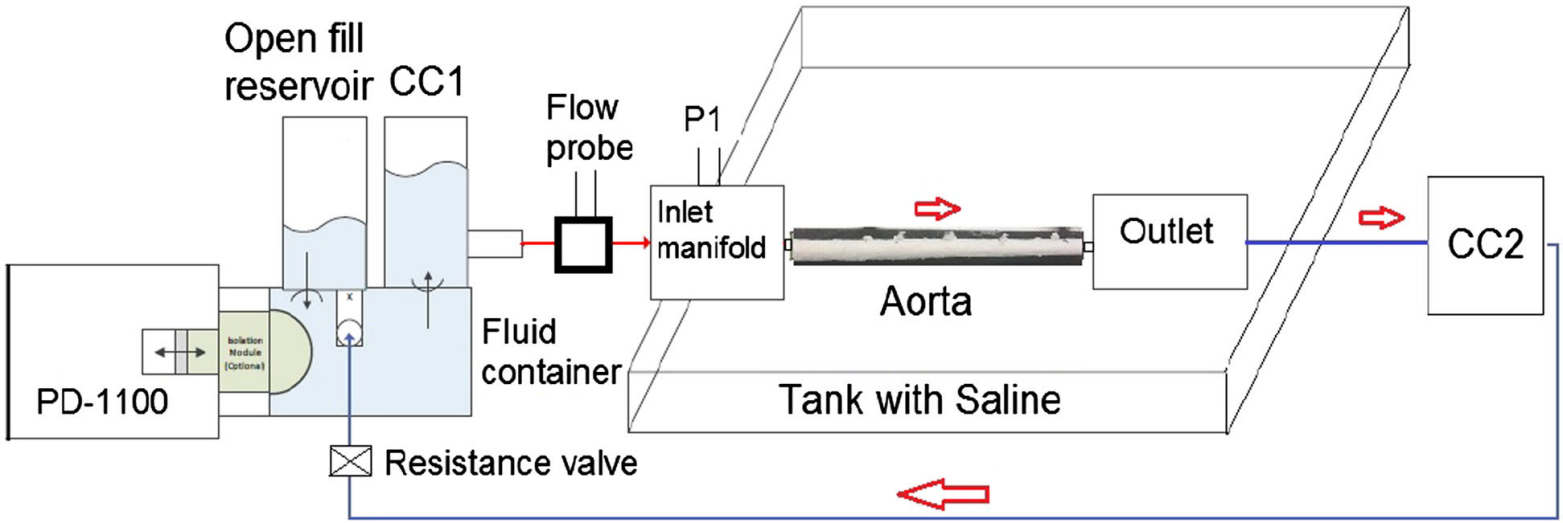
Schematic of the experimental set-up showing the major components. PD-1100 is the pulsatile flow pump. CC refers to the compliance chamber. P1 labeled at the inlet manifold is the site for inlet pressure measurements.

The pulse duplicator (PD; BDC laboratories, Wheat Ridge, CO, USA) set-up consisted of an electronically actuated pulsatile flow pump controlled by software (Statsys PD, BDC laboratories, Wheat Ride, CO, USA). This pump can be used to control the input waveform, heart rate, %systole and diastole, and mean flow rate. The pump consists of an isolation chamber to avoid contact with the test fluid. The isolation chamber is connected to an input reservoir chamber (Fig. 1) and an attached compliance chamber (CC1). The outlet from CC1 is connected to a rectangular container tank with saline, where the inlet and outlet ports can be used to mount the porcine aorta. The outlet is connected to a downstream compliance chamber (CC2), which then loops back to the inlet reservoir *via* a resistance valve (Swagelok, Solon, OH, USA).

An inline Transonics flow probe (ME13PXN; Transonics Inc., Ithaca, NY) was connected between the CC1 and container tank to measure the inlet flow rate. The mean pressure in the system was controlled using the resistance valve. The pulse pressure in the system was increased by heightened fluid level in the CC1 and vice versa. The CC2 acts as a downstream capacitor and reduces the negative component in the flow pulse. Blood mimicking fluid (Computerized Imaging Reference Systems, Inc. Norfolk, VA) was used as a blood analog, with a *ρ* = 1050 kg/m^3^, and dynamic viscosity, *μ* = 0.004 Pa s.

### Vessel Preparation

The tissue collection and animal sacrifice were performed as per the regulations set forth by the Institutional Animal Care and Use Committee. Descending thoracic porcine aorta (*n* = 36), ~20 cm in length were obtained on the day of sacrifice, either from local slaughterhouse (*n* = 30; Sierra for medical science, Whittier, CA, USA) or in-house acute studies (*n* = 6). The *in vivo* length of the aorta was measured between two points, marked using carbon black particles before sacrificing the pig. The descending thoracic aorta was then harvested and the vessel was stretched to the *in vivo* length based on the marked points. On an average, a stretch ratio of 30% was found to match the *in vivo* length similar to previous studies.^15^,^17^,^19^ The same stretch ratio was used for the aortas obtained from slaughter house. The vessel preparation was finalized by removing any loose adventitial tissue and ligating the major branches of the aorta with silk suture.

### Dissection

**Figure 2:**
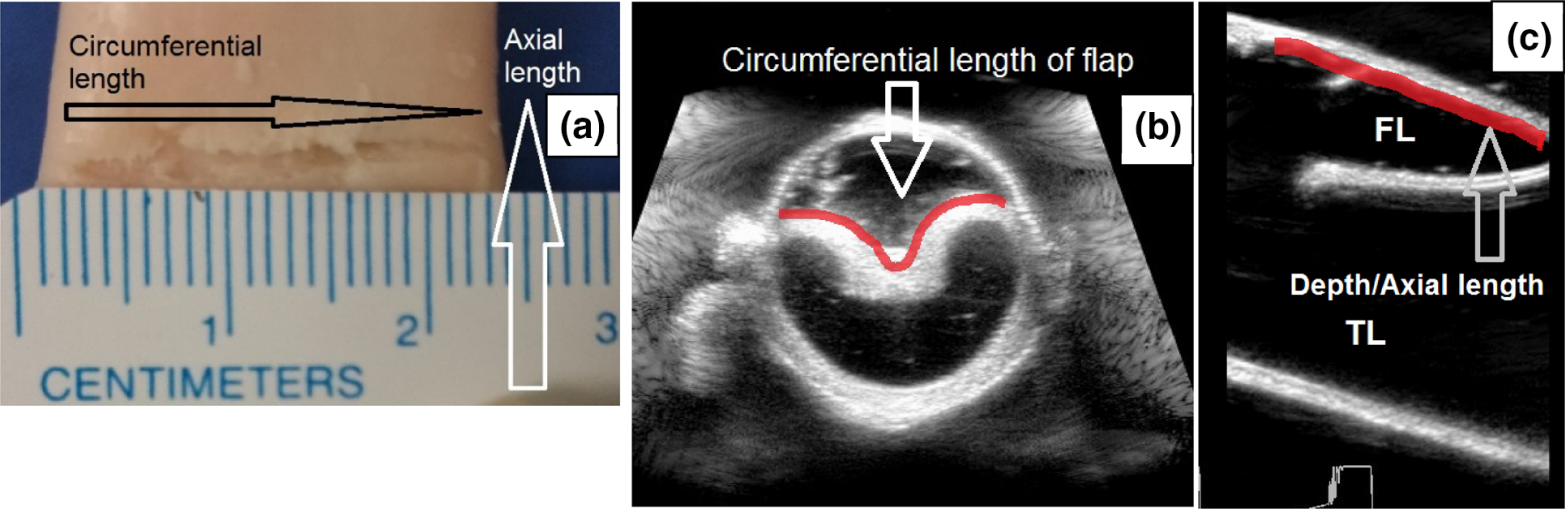
(a) The figure shows a dissection created in an inverted porcine aorta. The circumferential length and axial length (depth) of the dissection are shown. (b) Circumferential dissection as seen using ultrasound imaging. (c) Axial length of the flap as seen through ultrasound imaging

**Figure 3:**
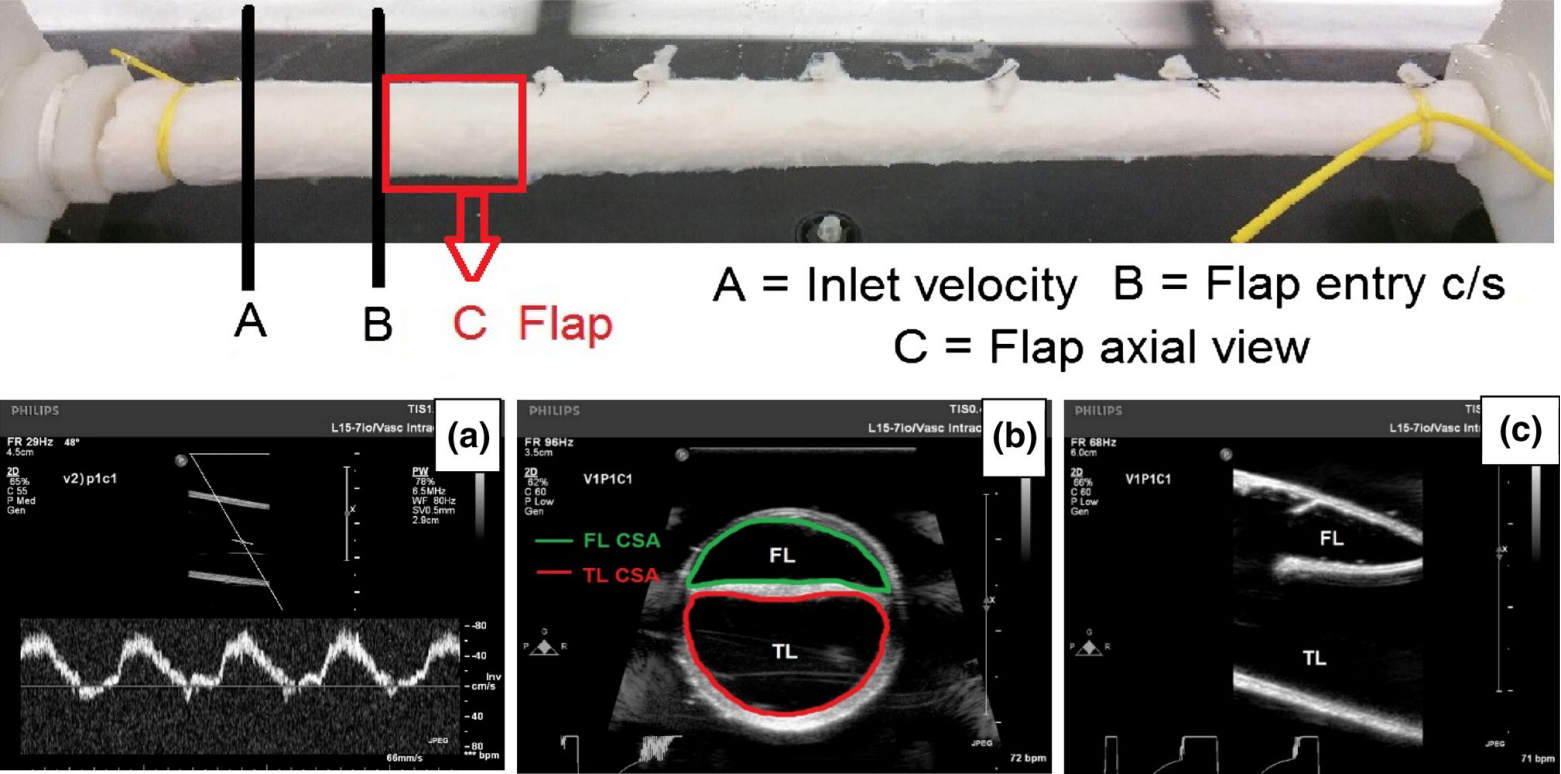
(a) Inlet velocity, (b) cross-sectional view of the TL and FL, (c) axial view of the TL and FL, (d) velocity in the FL, and (e) Velocity in the TL. All panels at 120/80 mmHg pressure.

The aorta was inverted exposing the intima and the dissections were created ~6–8 cm from the left subclavian artery. The inversion was performed carefully by holding the two ends of the aorta with forceps. In the experimental set-up, these two ends were placed over the connector and tied in place (Fig. 3). Other than the two ends held using the forceps, no other part of the aorta was affected by the inversion process. The % circumferential length of the entry tear (Fig. 2) was calculated as 100× (circumference of the flap/circumference of the vessel). Using a surgical blade, a cut was made in the intima-medial layers of the vessel. The circumferential length of the entry tear was measured (Fig. 2) and later confirmed using ultrasound (US) measurements. The intima-medial layers were separated using a surgical blade and advanced to the desired axial length (Fig. 2). The aorta was then inverted back. In this way, a single entry FL without an exit tear was created in the vessel wall.

### Hemodynamics

The pump input parameters were fixed at heart rate (HR) of 72 bpm and systole/diastole ratio = 35/65%. The pump flow rate was adjusted to achieve an inlet flow rate of ~2 L/min, at a max/min pressure of 120/80 mmHg. This flow rate provided a dynamic similarity with physiological velocity profiles found in descending aorta^33^ based on Reynold’s and Womersely numbers.^11^,^20^,^22^ The input velocity was measured using pulse wave (PW) US at approximately the centerline of the vessel. The mean Reynold’s number (Re) at the PD inlet used was 1134 (mean velocity = 0.27 m/s), and the peak Re was 3234 (maximum velocity = 0.77 m/s), based on the ultrasound velocity reading near the inlet, with a Womersely number of 11.3. There values are in agreement with the values reported in the descending human aorta, with a mean Womersely number of ~12.5 ± 1.5, mean Re number value of ~1015 ± 201, and a peak Re number value of ~3357 ± 590.^33^

### Study Protocols

For all the test cases, the inlet pressure was set at ~120/80 mmHg with a pulse pressure of 40 mmHg, and mean pressure of 100 mmHg. The pulse pressure was varied starting from 40 to 200 mmHg, in increments of 20 mmHg. Two mean pressures were tested, 100 and 180 mmHg.

For the flap geometry studies, the entry tear was characterized by the thickness of the flap, %circumference of the entry tear and axial length (or depth) of the flap. Two thicknesses were considered: Thin flap (1/3rd the thickness of vessel wall, *n* = 25 from 18 vessels) and a thick flap (2/3rd the thickness of the vessel wall, *n* = 25 from 18 vessels). The %circumference of the entry tear was varied between 15 and 65%, in increments of 10%. The axial length was varied from 0.5 to 3.2 cm, in increments of 0.5 cm. Only a single dissection was created in each vessel. A propagation was defined as both the circumferential and axial increase in the size of the initial tear.

For the flap hemodynamic (mean and pulse pressure) studies, the thick dissection flap with a fixed entry tear geometry (thick flap, axial length ~1.5 cm) were used. At each mean (100 and 180 mmHg) and pulse pressure (40, 60, 80, 110 mmHg and right before propagation), images of the CSA area of the entry tear (Fig. 3b) were acquired. Nine vessels were used to obtain 6 data points for each mean pressure and pulse pressure.

### Data Acquisition

For all the test conditions specified in the study protocol section, the inlet and the outlet pressures (Fig. 1), and the inlet volumetric flow rate were measured (Fig. 1) using the Statsys PD integrated data-acquisition system (BDC laboratories, Wheat Ridge, CO, USA). The pressure and flow data was obtained for 5 s at 5000 Hz frequency.

The flap characteristics and velocities were measured using a L15-i70 ultrasound (US) transducer connected to iE33 ultrasound machine (Phillips Ultrasound, Bothell, WA, USA). The vessel and flap dimensions (Figs. 2b and 2c), change in cross-sectional area (CSA) over the cardiac cycle (Fig. 2b), and CSA of the flap was recorded in B-mode. The ECG leads on the US machine were synchronized with the pump activation to obtain the %cardiac cycle at which flap measurements were made. The pressure values from the inlet manifold (Fig. 1) were obtained on the Statsys PD software. The %cardiac cycle values obtained from the pump actuation signal were used to obtain the corresponding pressure values from the Statsys PD pressure signal.

The velocities were measured in the pulse wave Doppler mode, by adjusting the corrected steer angle in-line with the flow. At the pump settings described above, an aortic velocity waveform was achieved. This is apparent from the velocity measured at the inlet of the vessel, 3 cm proximal to the flap near the connector (Fig. 3a).

### Data Analysis

The images from ultrasound were transferred to ImageJ (NIH, Bethesda, MD, USA) for further analysis. Images with noise were converted to binary (OTSU method) and the edges were detected using the “find edges” option under the process toolbar available in ImageJ. The diameter of the vessel at the entry was also measured. The TL and FL cross-sectional area were traced (Fig. 3b) using a free hand tool and the area was quantified. The calibration values in units of cm were obtained from the DICOM tags of the US images. The CSA were quantified at ~8 points during a cardiac cycle (acquisition rate = 30 fps; 1 cardiac cycle = 1.2 s). The %TL CSA was quantified as:

$$ \% {\text{TL CSA}} = 100 \times \left[ {{\text{TL CSA}}/({\text{TL CSA}} + {\text{FL CSA}})} \right]. $$

During a cardiac cycle, the %max change in TL area was calculated as: 100 × (%TL_CSA max_ − %TL_CSA min_)/%TL_CSAmax_). Similar calculations were performed for the FL.

The energy acting in the FL during propagation was calculated. Under the assumptions described in Appendix, the maximum work done during a cardiac cycle in the FL can be determined as the difference between the product of the maximum pressure and the corresponding volume and the product of the minimum pressure and its corresponding volume during the cardiac cycle: [(*p* × *v*)_max_ − (*p* × *v*)_min_]. The pressure values at the respective cardiac cycle were obtained from pressure recordings of the pulse duplicator. The volume (v) of the FL was computed by approximating the FL to a cone with an elliptic/circular base (i.e., $v = \frac{\pi }{3} CSA \times h$ where h is the length of the FL wall). In order to standardize the comparison, the de-cohesive energy/unit area (J/cm^2^; area of the c/s of the entry tear) was used.

### Statistical Analysis

All the data is presented as mean ± SD. Comparisons were performed using a paired, two-tailed student’s *t* test. Regression analysis was performed to assess the trend. All the analyses were performed using Medcalc (Ostend, Belgium). *p* < 0.05 was considered statistically significant.

## Results

### Relation Between Geometric Parameters and Flap Propagation

**Figure 4:**
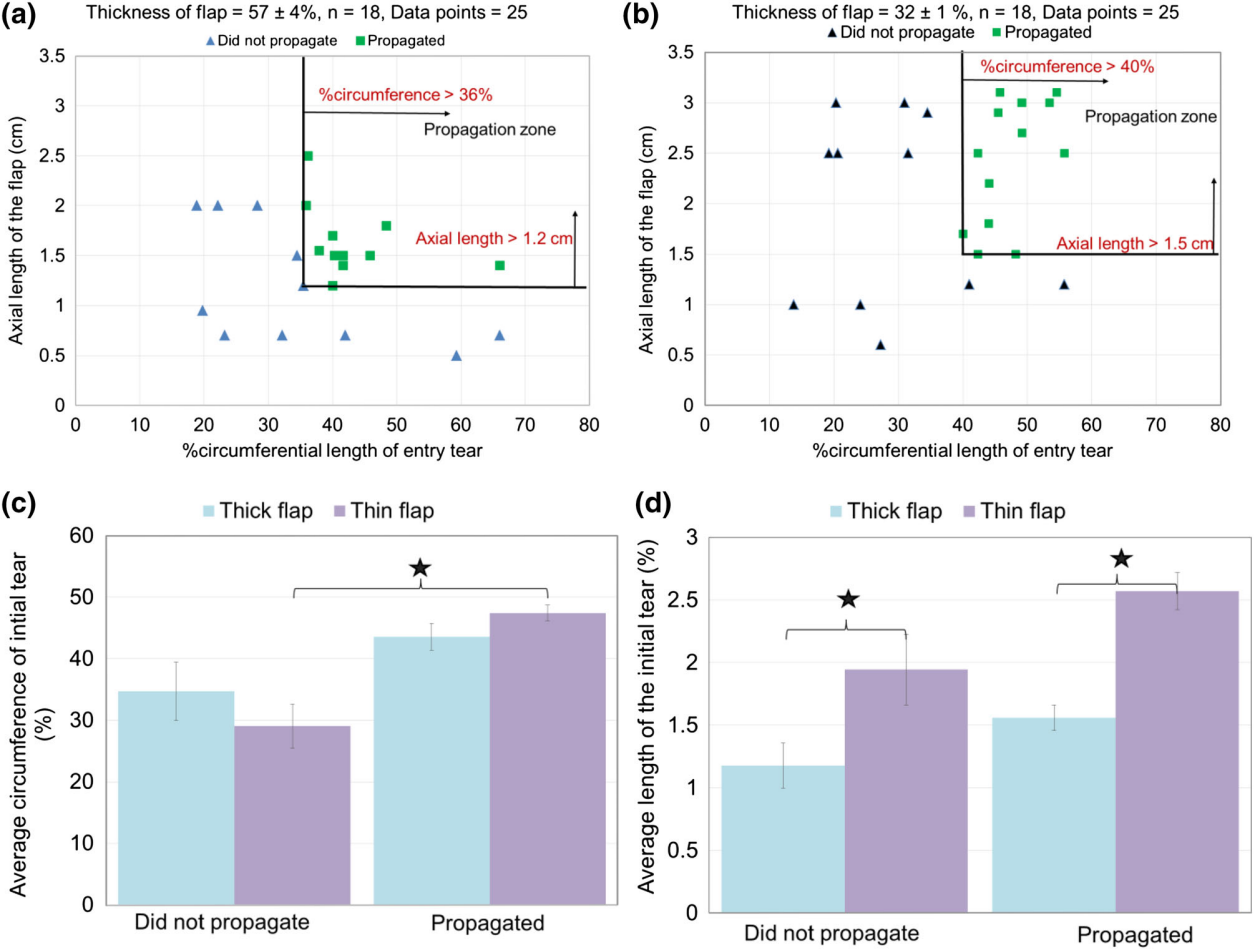
Relation between axial length and %circumference of the entry tear for (a) thick flap, (b) thin flap A vessel was marked as “did not propagate” or “propagated” for the tested pressure ranges of 180 mmHg and a pulse pressure of 200 mmHg. (c) Summary of comparisons of the average %circumference values for the propagated and non-propagated regions for the thick and thin flaps. (d) Summary of comparisons of the average axial length of the propagated and non-propagated regions for the thick and thin flaps. The values are presented as mean ± standard error. The star symbol with a *p* < 0.05.

The relation between %circumferential length of entry tear, and axial length of the flap for a thick flap and thin flap are presented in Figs. 4a and 4b, respectively. For a thick flap (Fig. 4a; 57.1 ± 4.25%), a propagation occurred for the flaps with %circumference of the entry tear >36%, and axial length >1.2 cm. For a thin flap (Fig. 4b; 31.9 ± 1.42%), tears with a %circumference >40% and axial flap length >1.5 cm resulted in a propagation under tested conditions. Similar to thick flap, the flaps below the geometric threshold did not propagate below a mean pressure of 180 mmHg and pulse pressure of 200 mmHg. In addition, flaps with circumference >40% and depth >2.5 cm propagated readily (Fig. 4a) when the pump was initiated, due to complete obstruction of TL.

A summary of the average values and significant comparisons for the propagated and non-propagated thick and thin flaps is provided in Figs. 4c (%circumference values) and 4d (axial length values). On an average, for the thin flap propagation occurred in flaps with %circumference of 47 ± 1 which was not significantly different (*p* > 0.05) from the %circumference (44 ± 2) of the propagated vessels with a thick flap (Fig. 4c). On the other hand, for the thin flap propagated vessels had an axial length of 2.57 ± 0.15 cm which was significantly different (*p* < 0.05) from the axial length (1.56 ± 0.1) of the propagated vessels with a thick flap (Fig. 4d).

In Fig. 4a, the mean propagation pressure values were within physiological range (mean of 100 and 180 mmHg, with pulse pressure ranging from 100 to 200 mmHg). Based on specific geometric boundaries of dissection, the flap dynamics were studied in relation to pressure and energy required for propagation as described below.

### Relation Between Flap Movement and Mean/Pulse Pressure

**Figure 5:**
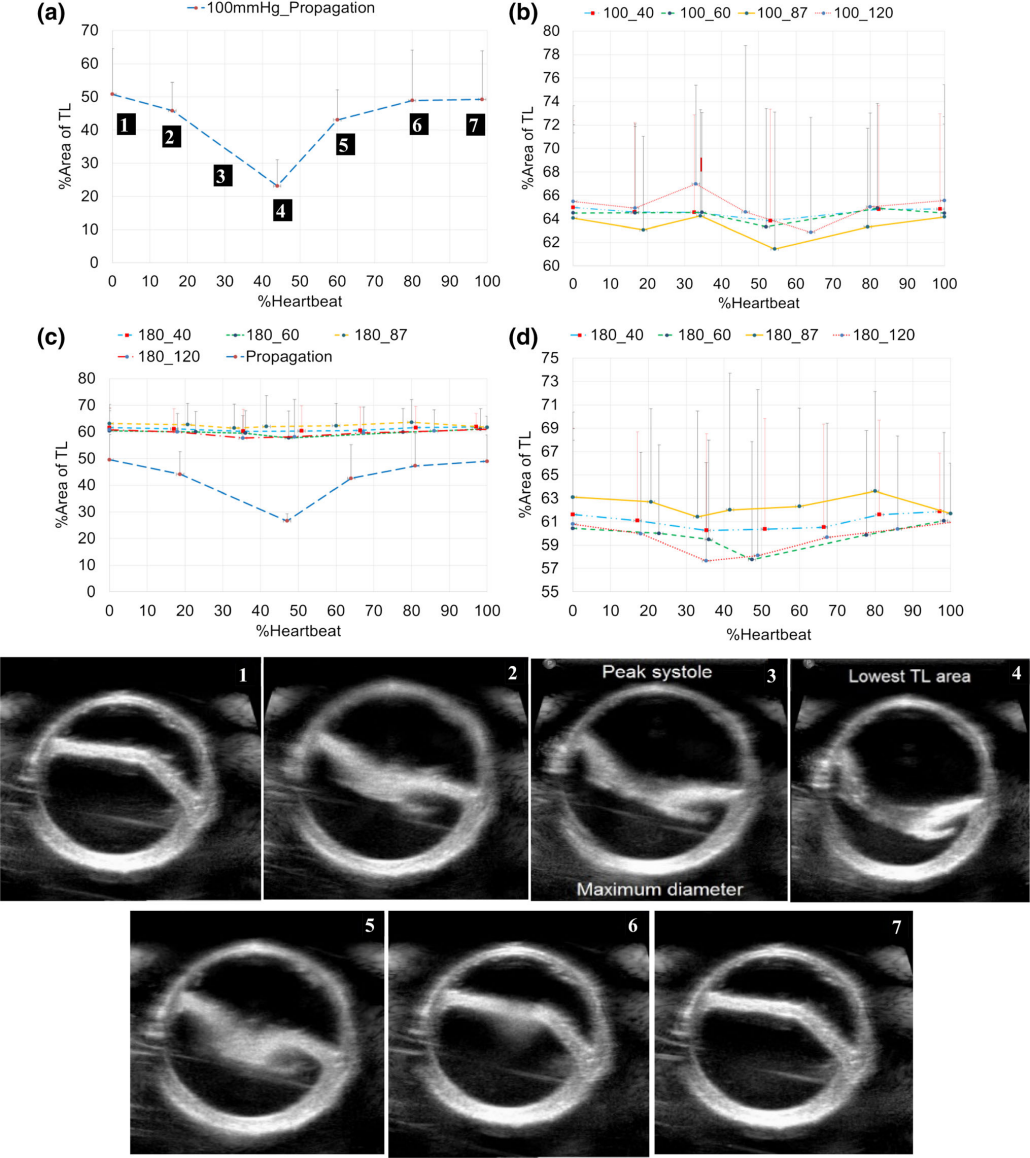
Flap movement during cardiac cycle, at a given mean pressure with changing pulse pressure (*n* = 6). The curves were generated from an averaged data set from 6 vessels. Each data point represents the mean value ± SD for both the *x*- and *y*-axes values. (a) Propagation curve for a mean pressure of 100 mmHg. (b) Non propagation curves for a mean pressure of 100 mmHg. The legend of the figures represents the mean pressure_pulse pressure. 100_40 represents a mean pressure of 100 mmHg and a pulse pressure of 40 mmHg. (c) Propagation curve for a mean pressure of 180 mmHg. (d) Non propagation curves for a mean pressure of 180 mmHg. Legend 180_40 represents the mean pressure_pulse pressure.

For the vessels with thick flap (57 ± 4%) and a fixed geometry of entry tear (%circumference = 39 ± 2%; axial length = 1.43 ± 0.13 cm), the relationship between the flap dynamics and the mean and pulse pressures was determined. Figure 5a summarizes the change in the %CSA of the TL during a cardiac cycle, for a fixed mean pressure of 100 mmHg, under increasing pulse pressures. Each curve represents the %change in TL area during a cardiac cycle for pulse pressures of 40, 60, 80 and 110 mmHg. Further, the flap movement immediately before propagation is also presented. It can be observed that the peak reduction in the TL occurs during the systolic phase, slightly after the peak systole (~ at 45% of the cardiac cycle). Similar changes in %area of TL were observed for a mean pressure of 180 mmHg (Fig. 5b), under varying pulse pressure values.

The flap movements during the cardiac cycle are presented in the panels of Fig. 5. At the start of the cardiac cycle (panel 1 corresponding to the point A in Fig. 5a), the flap was curved towards the FL. At 18% of the cardiac cycle (panel 2), the vessel began to increase in diameter and the flap was pushed towards the TL. At peak systole (panel 3: 35% of cardiac cycle), maximum vessel diameter was observed, and the flap was further pushed towards the TL. Slightly after, at 45% of the cardiac cycle (panel 4), the vessel diameter began to decrease while the flap was further pushed towards the TL, resulting in the lowest TL CSA during the cardiac cycle. The flap began reverting to its original position as the cardiac cycle progressed, as shown in panel 5 (60%), panel 6 (80%), returning to the original position at the end of cardiac cycle (panel 7).

**Figure 6:**
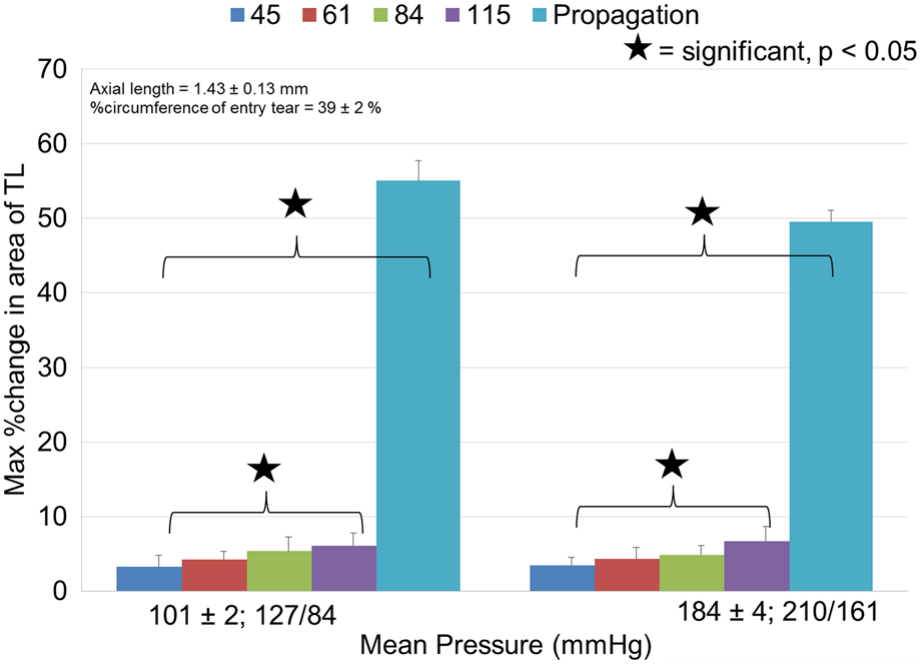
Comparison of maximum %change in CSA of TL during a cardiac cycle under different pulse pressures (*n* = 6). The legend represents the different pulse pressure values in mmHg. The *x*-axis titles represent the mean pressure values, 101 mmHg and the corresponding systolic (127 mmHg) and diastolic (84 mmHg) pressure values, similarly the mean pressure of 184 mmHg with a systolic pressure of 210 mmHg and diastolic pressure of 161 mmHg

To quantify these observations, the %variation in TL area and the max %change in the flap during a cardiac cycle was computed as shown in Fig. 6. As the mean pressure varied from 100 to 180 mmHg (Fig. 6), there was no difference in the variation of the %CSA of the TL, for all the tested pulse pressures (45 mmHg = 6%, *p* = 0.84; 61 mmHg = 4%, *p* = 0.79; 85 mmHg = 8%, *p* = 0.63; 116 mmHg = 10%, *p* = 0.49). The same trend persisted for the flap movement right before propagation (11%, *p* = 0.06).

On the contrary, for a fixed mean pressure (Fig. 6), there was a significant increase in the flap moment with a change in pulse pressure (100 mmHg = 85%, *p* = 0.02, 180 mmHg = 92%, *p* = 0.003). On average, propagations occurred at a pulse pressure of 159 ± 42 mmHg. At these pulse pressures, immediately before propagation (52 ± 4%), the %change in the TL area was significantly increased (*p* < 0.0001) in comparison to all previous pulse pressures (5 ± 1%).

### Work Done and the De-cohesive Energy

For all the vessels with similar tear geometry, and simultaneous pressure and TL entry CSA measurements, the total work done or the de-cohesive energy acting in the FL was computed (*n* = 7), right before the propagation occurred. The mean value of de-cohesive energy was 0.047 ± 0.010 J. The amount of de-cohesive energy acting on the FL before propagation remained similar (*p* < 0.05) irrespective of the mean pressure. The mean de-cohesive energy per unit area of FL was 75 ± 9 J/m^2^.

## Discussion

This is the first study to describe a realistic ex vivo model for simulation of aortic dissection to understand the relationship between flap dynamics and pulse pressure. The major findings of this study are as follows: (1) Pulse pressure rather than mean pressure is the primary determinant of the flap movement and hence flap propagation, and (2) Geometry of the initial intimal-medial tear (i.e., the circumferential, axial length and depth) has a significant influence on flap propagation.

### Implications of the Role of Pulse Pressure

In the clinical environment, mean pressure is closely monitored in patients who are diagnosed with aortic dissection, to prevent further propagation. This study suggests that pulse rather than mean pressure may be more of a culprit for dissection propagation. The major risk factors for aortic dissection are known as atherosclerosis (due to ageing), hypertension, trauma, and congenital/genetic diseases. In addition to reduced vessel wall strength, the common factor in these risk factors is the variation in pulse pressure. Furthermore, pulse pressure (but not the mean pressure) has been shown to be associated with adverse cardiac outcomes in older patients.^1^,^8^ For a given mean pressure, higher pulse pressure is believed to lead to arterial damage^1^,^8^ which is also thought to be an important predictor of cardiovascular mortality^7^,^16^,^24^ in some patient outcome studies.^24^

Our results show that pulse pressure greatly impacts the mobility of the intimal-medial flap and contributes to propagation (Fig. 5). As pulse pressure increases, there is a gradual increase in flap movement followed by a sudden jump before propagation occurs. With aging and arteriosclerosis, pulse pressure is known to increase as arterial stiffness increases.^7^,^16^ It is interesting to note that control of hypertension, which is thought to be a major risk factor for aortic dissection, is also characterized by increased pulse pressure. Clinically, pulse pressures of the order of ~90 mmHg or more have been observed in patients with acute aortic dissections.^12^ Further studies will be required to assess the clinical benefit of preferentially controlling pulse pressure rather than mean pressure in patients with aortic dissection.

Other events like increased mental and physical stress^35^ and extreme exercise conditions^31^ may lead to transient elevations in pulse pressures. More importantly, blunt trauma can lead to temporary loss and sudden restoration of pulse pressure. These “trigger” events could lead to a temporary onset of increased pulse pressures and de-stabilized flap movement potentially leading to dissection propagation.

### Flap Movement and Characteristics

The propagation of a dissection is an abrupt and acute phenomenon which is rare to observe clinically. Hence, model studies focusing on dissection propagation may lead to insights into the role of flap dynamics of a vessel before dissection propagation and provide guidance for interventions. For example, in the majority of vessels tested in this study, the flap curved towards the TL rather than the FL before the propagation of a dissection. Under *in vivo* conditions, the arteries are in a stretched configuration. A disruption in the layers of the arterial wall leads to a slack in the middle portion of the flap in comparison to the edges, which are attached to the wall. This slack in the circumferential length of flap leads to a “tongue” with a curvature towards the opposite wall. Under normal conditions, the “tongue” of the FL, at its maximum displacement (occurring a little after peak systole, Fig. 5) did not lead to a significant obstruction of the TL (~ 10% reduction in TL CSA). Under propagation conditions, the flap movement increased considerably (52 ± 4%). The propagations were instantaneous and occurred in both the circumferential and axial directions. After propagation, the flap was curved towards the FL and caused a complete obstruction of the TL during the systolic phase.

The blood supply to the organs occurs primarily during systole. If the branch vessels supplying blood to major organs originate from the TL, the obstruction of TL during systole may lead to loss of blood supply to end-organs. If the branch vessels originate in the FL, the amount of blood supplied may be limited. This phenomena may explain the high mortality and complications arising due to acute dissection.

In literature, there are a few studies^2^,^12^,^38^ that have characterized the flap motion with cardiac cycle for chronic and acute dissection cases. Of these, there was one study that explored the movement in acute dissections.^38^ Yang *et al.* (2014) in 49 patients,^38^ investigated the flap movement in the top 2 cm of the celiac trunk ostium. This study reported that there was a flap movement of 49.5% ± 23.5% during a cardiac cycle. This is in line with the flap movement observed in the present study (52 ± 4%).

Based on these observations, a clinical recommendation would be to restrict the flap movement. This could be achieved by use of stent grafts or by mitigation of clinical factors (for example, pulse pressure) responsible for increased FL movement.

### De-cohesive Energy

Using mechanical peeling tests, Sommer *et al.*,^32^ reported the dissection energy for normal (no significant pathological changes) human abdominal aorta as 51 ± 6 J/m^2^ in the circumferential direction and 76 ± 27 J/m^2^ in the axial direction. In this study, the average energy acting on the FL right before propagation was 75 ± 13 J/m^2^. This energy is a sum of the elastic energy stored in the flap, energy required for the movement of the flap and the FL walls, and the energy leading to de-cohesion of the aortic wall. This value is in close agreement with the values reported by the above study. It should be noted that the current study used healthy porcine tissue and the de-cohesive energy is very likely affected by the disease state of the aorta. Using hydro-dissection models, under diseased condition (atherosclerosis) of human aorta,^34^ another study reported a much lowered de-cohesive energy of 1.65 J/m^2^ for medial separation.

In porcine tissue, the de-cohesive energy for medial separation was reported with a wider variation. In the upper descending thoracic aorta, one study^4^ reported a value of 159 ± 9 J/m^2^ while another study reported 28.4 ± 12 J/m^2^ (upper thoracic),^29^ and 29 ± 12 J/m^2^ (lower thoracic).^29^ Similar wide variations were reported in the upper abdominal aorta^29^ as 18.8 ± 8.9 mJ/cm^2^, and 113.4 ± 40.5 mJ/cm^2^ for the lower abdominal aorta.^29^ These energies are reported for the initiation and propagation of a bleb as opposed to the propagation only explored in the current study.

### Assumptions and Limitations

This study modeled the propagation of aortic dissection along a linear stretch of descending thoracic aorta, as is seen with Type B dissections. The morphology of the aortic arch was not accounted for in these experiments. Additionally, the descending aorta might also have significant tortuosity, particularly in older patient population. In addition to the factors explored in this study, tortuosity may also play a role in the tear initiation and propagation. This effect needs to be explored in further studies.

The effect of branch vessels was not included in the current study. The initial entry tear was created in a region without branch vessels. A previous study has shown that branched vessels affect the peripheral resistance and pressure values depending on whether they are branching off the TL or FL.^6^ Despite the ligation of branch vessels, however, physiological pressure values and waveforms were achieved in the test set-up. In addition, dynamic flow similarity was achieved based on velocity waveforms. Hence, we believe the results of this study would be largely unaffected. Aortic dissection consists of an initiation phase followed by a propagation phase. This study focuses on the factors responsible for propagation, assuming that the initiation has already occurred. Furthermore, we studied the flap dynamics in healthy porcine aorta. It is likely that a smaller intimal-medial injury and lower mean and pulse pressures are required for propagation of such dissection in atherosclerotic diseased human aorta. In chronic dissection, endothelialization and remodeling of the false lumen can occur which were not considered in the current study. Finally, spontaneous intramural hematoma is believed to be a related process that may lead to aortic dissection, but the relationship with this entity and frank aortic dissection was not been explored here.

The de-cohesive energy was computed based on the inlet pressure rather than a direct measurement of the pressure within the false lumen. The dynamic nature of the flap and abrupt incidence of propagation precluded the direct measurement of FL pressures during propagation. Further, there would be an in-and-out movement of the fluid inside the FL over the cardiac cycle. This leads to a spatially non-uniform pressure within the FL even at a single fixed time. Another assumption in the energy calculations is the approximation of the FL to a cone with an ellipsoid base. The FL geometry is more complicated and spatially non-homogeneous. These non-homogenous effects are hard to quantify in an *in vitro* setup and are considered a limitation of the current approach.

In literature, the tear size and location were reported to impact the FL pressures in chronic dissection.^36^ The flap length tested in this study was 1.5 cm, however, which is very small in comparison to the 24 cm flap used in our study. Hence, we believe this is a reasonable approximation.

## Conclusions

Flap propagation was dependent upon initial geometry of the entry tear for both the thick and thin flaps. Pulse pressure had a significant effect on the flap movement in contrast to mean pressure. Hence, mitigation of pulse pressure and restriction of flap movement may be beneficial in patients with type B acute dissections.

## Appendix

Under assumptions of steady state propagation, the work done for the dissection propagation can be written as a sum of the change in elastic energy of the flap (Δ*W*_flap_), and the de-cohesive energy per unit area ($\bar{E}$) times the change in area (Δ*A*) as

1 $$ E = \Delta W_{\text{flap}} + \bar{E}\Delta A, $$

where *E* is the total de-cohesive energy for flap propagation; $\bar{E}$ = De-cohesive energy per unit area, $= \frac{E}{A};$ and Δ*W*_flap_ is the elastic energy of the flap.

The analysis was carried out under the following assumptions: (1) the flap is fully stretched such that there is minimum increase of strain when dissection propagates. (2) Δ*W*_flap_ is very small compared to $\bar{E}\Delta A.$ (3) The propagation occurs circumferentially, and then along the axis (axial length or depth) of the vessel. (4) The pressure is equally distributed in all the directions. (5) The FL has approximately the same shape along the depth (axial length) of the flap, just becomes longer.

However, in a bench-top set-up, *E* can be computed from the change in the pulse pressure and the change in the corresponding volume of the false lumen (FL), as detailed below. The flap can be approximated as a cone with ellipsoid base (Fig. 2). The assumptions are as follows: (1) The tongue shape of the entry tear persists throughout the flap depth. (2) The stretch in the depth of the flap has negligible effect on the volume. (3) There is uniform stretch of the flap in all the directions. (4) The pressure inside the false lumen could be approximated to the inlet pressure.

2 $$ E = \Delta {\text{W}}_{\text{flap}} + \bar{E}\Delta A = \Delta P \times \Delta V_{\text{FL}} , $$

where Δ*P* is the pressure differential, or the pulse pressure defined as the difference between the systolic and diastolic pressure; Δ*V*_*FL*_ is the change in volume of the false lumen.

Based on the above, the volume of the flap can be computed as [(*π*/3) × CSA^2^ × *h*]; where CSA = *π* × *a* × *b*, for an ellipse, *a* = major axis; *b* = minor axis of the ellipse (cm); *h* = height of the cone, assumed to be equal to the depth of the flap in this case. In order to minimize the error due to fitting an ellipse, direct measurement of the cross-sectional area of the entry tear (CSA = *π* × *a* × *b*), measured using ImageJ, was used in the above formula. Hence, the volume of the false lumen is a function of the geometry of the entry tear, the %circumference of the entry tear (%C) and the axial length of the flap (*D*). Furthermore, the work done during a cardiac cycle leading to the change in volume (∆*V*) is dependent upon the pulse pressure and the geometric parameters. Accordingly, the decohesive energy can be estimated as the difference between the maximum and minimum product of pressure and volume of the flap,

$$ E = \left[ {(p \times v)_{ \hbox{max} } {-}(p \times v)_{ \hbox{min} } } \right]. $$
